# Fluoride Content in Alcoholic Drinks

**DOI:** 10.1007/s12011-015-0519-9

**Published:** 2015-10-16

**Authors:** Marta Goschorska, Izabela Gutowska, Irena Baranowska-Bosiacka, Monika Ewa Rać, Dariusz Chlubek

**Affiliations:** Department of Biochemistry, Pomeranian Medical University, PowstańcówWlkp. av. 72, 70-111 Szczecin, Poland; Department of Biochemistry and Human Nutrition, Pomeranian Medical University in Szczecin, Broniewskiego 24 street, 71-460 Szczecin, Poland

**Keywords:** Alcoholic drinks, Fluoride content, Fluoride intake

## Abstract

The aim of the study was to determine the role of alcoholic drinks as a potential source of dietary fluoride by means of measuring fluoride levels in selected alcoholic drinks available on the Polish market that are also diverse in terms of the percentage content of ethanol. The study was conducted on 48 types of drinks with low, medium, and high alcohol content available on the Polish market and offered by various manufacturers, both Polish and foreign. Fluoride concentrations in individual samples were measured by potentiometric method with a fluoride ion-selective electrode. The highest fluoride levels were determined in the lowest percentage drinks (less than 10 % *v*/*v* ethanol), with the lowest fluoride levels observed in the highest percentage drinks (above 40 % *v*/*v* ethanol). In terms of types of alcoholic drinks, the highest fluoride levels were determined in beers and wines, while the lowest levels were observed in vodkas. These data confirm the fact that alcoholic beverages need to be considered as a significant source of fluoride delivered into the body.

## Introduction

The human body is constantly exposed to fluoride due to the consumption of food products, the main source of this element for humans [1, 2]. A significant part of this ingested fluoride is contained in beverages, including alcoholic drinks, a quantitatively significant component of the diet of many people and the primary source of exogenous ethyl alcohol [3]. Because of their prevalence and volume of consumption, they should be considered a potential source of other xenobiotics and taken into account in preparing a balanced diet [4]. Due to the continuing controversy over the use of fluorine compounds in the prevention of tooth decay and the possible adverse effects (fluorosis), it seems prudent to examine the presence of fluoride in common food products, including alcoholic beverages [5]. The small number of publications on the amount of fluoride in alcoholic beverages has led us to examine them as a possible source of fluoride in the diet.

The aim of the study was to determine the role of alcoholic drinks as a potential source of dietary fluoride by means of measuring fluoride levels in selected alcoholic drinks available on the Polish market that are also diverse in terms of the percentage content of ethanol.

## Material and Methods

### Characteristics of Samples

The study was conducted on 48 types of drinks with low, medium, and high alcohol content available on the Polish market and offered by various manufacturers, both Polish and foreign. The examined beverages were classified according to the percentage of ethyl alcohol:

1. <10 % of ethanol
2. 10–20 % of ethanol
3. 20–40 % of ethanol
4. > 40 % of ethanol

Moreover, the alcoholic beverages tested for fluoride content were also classified in terms of the production process, namely: (1) vodka, (2) spirits with a high content of ethanol other than vodka, (3) rums, (4) liqueurs, (5) wines, and (6) beers.

### Determination of Fluoride Content in Collected Samples

Fluoride concentrations in individual samples were measured by the potentiometric method with a fluoride ion-selective electrode (Orion 9409 BN, Thermo Scientific, USA). Carbonated drinks were degassed before the measurements [6, 7].

Samples of alcoholic beverages were collected directly from bottles, after thorough mixing of the contents.

To a 0.5-ml solution sample, we added 0.5 ml of TISAB II buffer, and after 5 min the potential of the samples was measured by potentiometry using an ion-selective electrode. Then, 0.1 ml of the appropriate standard was added and measurement was performed again. The electrode had been calibrated using standard solutions.

According to the works of Gutowska et al. [8, 9], the fluoride content in samples was calculated based on the difference of potentials measured in each sample and the concentration of the added standard. The electrode had been calibrated using standard solutions.

### Statistic Analysis

For statistical analysis, nonparametric tests—Friedman ANOVA, Mann–Whitney *U* test, and Spearman test—were used. All calculations were performed using Statistica 10.0 software (StatSoft Poland). Values of *p* ≤ 0.05 were considered statistically significant. Since in most of the publications results are given as mean value, our results were expressed as median (values in Table 1) and mean ± SD (figures), so in the future those values may be used in various comparisons by other authors.

**Table 1:** Values of median and range of fluoride concentrations in the groups of drinks

Alcohol	Median	Lower quartile	Upper quartile
In dependence of percentage content of ethanol			
<10 %	0.131	0.103	0.172
10–20 %	0.098	0.093	0.160
20–40 %	0.056	0.034	0.110
>40 %	0.044	0.037	0.072
In dependence of various kinds of alcoholic drinks			
Vodkas	0.044	0.036	0.049
Color vodkas	0.050	0.041	0.073
Rums	0.045	0.030	0.118
Liqueurs	0.079	0.030	0.115
Wines	0.105	0.094	0.199
Bears	0.131	0.121	0.147

## Results

**Fig. 1:**
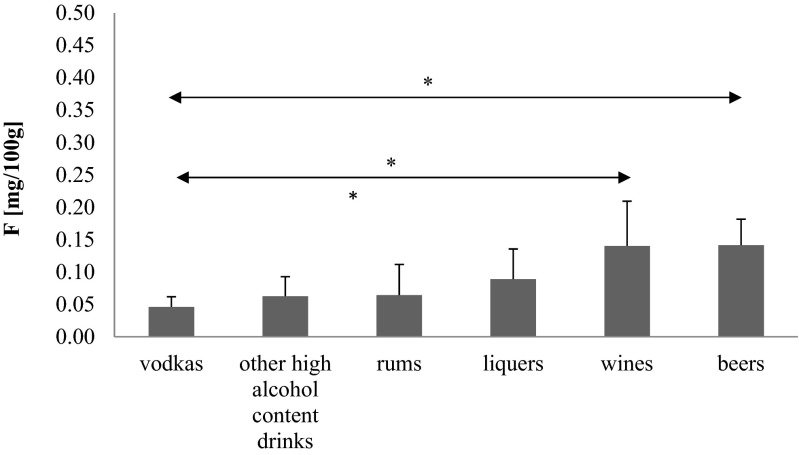
The content of fluoride in various kinds of alcoholic drinks. *p < 0.05 in comparison among each groups

The highest fluoride levels were determined in the lowest percentage drinks (less than 10 % *v*/*v* ethanol), with the lowest fluoride levels observed in the highest percentage drinks (above 40 % *v*/*v* ethanol) (Fig. 1).

The fluoride levels in the alcoholic drinks with less than 10 % and 10–20 % *v*/*v* ethanol were statistically significantly higher than in drinks with 20–40 % and above 40 % *v*/*v* ethanol (*p* < 0.05) (Fig. 1).

**Fig. 2:**
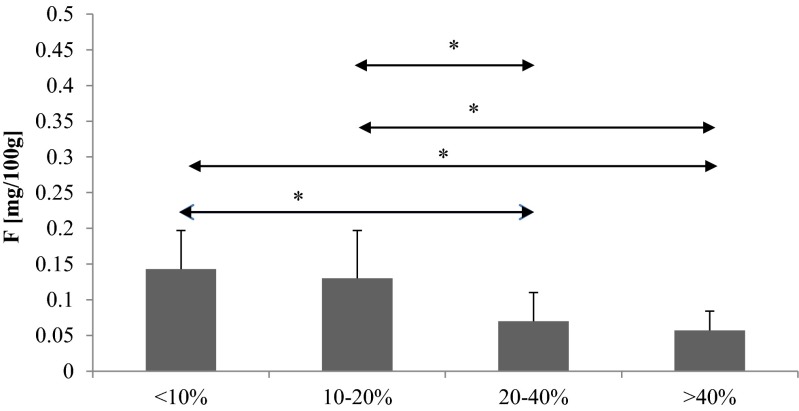
The content of fluoride in various kinds of alcoholic drinks in dependence of percentage content of ethanol. *p < 0.05 in comparison among each groups

In terms of types of alcoholic drinks, the highest fluoride levels were determined in beers and wines, while the lowest levels were observed in vodkas (Fig. 2).

Fluoride levels in both beers and wines were statistically significantly higher than the levels in vodkas (*p* < 0.05) (Fig. 2).

Spearman’s correlation analysis showed a negative significant correlation between the concentration of alcohol and fluoride content in beverages (rs = −0.67, *p* = 1.52 × 10^−07^).

## Discussion

According to the research of TNS OBOP (Polish Centre for Public Opinion Research), as many as 84 % of the adult population in Poland consume alcohol, the majority of which are men. On a European scale, those who admit to alcohol consumption account for 76 % of the population, while 24 % are abstainers. In 2012, an average of 9.25 l of alcohol was consumed per capita in Poland. Alcohol is most frequently consumed by people with higher education and urban residents, and beer is the alcohol of choice among Polish residents (87.6 %). Spirits are the second most widely consumed alcohol (7 %), followed by wine http://www.tnsglobal.pl/jakpijapolacy/pdf/raport.pdf.

In this study, alcohols from different brands available on the Polish market were examined. The fluoride levels correlated negatively with the levels of alcohol in beverages (rs = −0.67, *p* = 1.52 × 10^−07^). The highest concentration of fluoride was observed in the beverages with the lowest alcohol content (<10 %), while the lowest concentration was observed in the beverages with the highest percentage of alcohol. Having taken into account the type of alcoholic beverage, it became apparent that beer and wine had the highest levels of fluoride, while vodka and other spirits with a high content of alcohol had the lowest levels of fluoride http://www.tnsglobal.pl/jakpijapolacy/pdf/raport.pdf.

The United States Department of Agriculture (USDA) data from 2004 show the lowest fluoride levels in spirits (9 mg/100 g), including vodka, rum, gin, and whiskey http://www.ars.usda.gov/SP2UserFiles/Place/12354500/Data/Fluoride/F02.pdf. The same data shows higher levels of fluoride in regular beers (44 mg/100 g), lagers (45 mg/100 g), red wines (105 mg/100 g), and white wines (202 mg/100 g) http://www.ars.usda.gov/SP2UserFiles/Place/12354500/Data/Fluoride/F02.pdf. However, subsequent studies show that the level of fluoride can vary in one given type or even brand, for example depending on the place of production [10]. Beer brands Heineken and Carlsberg, with licensed production in the UK, may serve as an example: they have lower fluoride levels compared with those brewed in Denmark and the Netherlands [10]. The differences in fluoride levels in beers have been explained as an effect of the different levels of fluoride naturally occurring in water or water fluoridation in some regions [11, 12].

It is difficult to compare our results with other studies, mainly due to the lack of publications on such a large group of alcohols and a large spread of the percentage in the test material. However, the obtained results are consistent with data published by the USDA. As for the values of the fluoride concentration in low-alcohol beverages, our results are similar to those obtained by Rees et al. in a low-alcohol drink made on the basis of lemonade and ethyl alcohol [13] and the research on the concentration of fluoride in wines from the Canary Islands [12, 14].

Fluorine is a ubiquitous element in the natural human environment. It is well known that its intake at an appropriate level is necessary to prevent tooth decay. However, an excessive intake of fluoride can result in serious consequences for human health. It is estimated that the daily intake of fluoride in the UK is approx. 1.82 mg per day. It is noteworthy that more than 70 % of the fluoride delivered into the body every day is contained in beverages, especially tea [12]. The excessive ingestion of fluoride in foods and drinks leads, i.a., to erosion of the enamel and, in consequence, to fluorosis [15]. These effects are observed in people who live in endemic fluorosis areas in China [16]. Recently, it was pointed out that groups of people who regularly consume alcohol should be taken into special consideration. According to a study carried out in the UK, alcoholics can consume large volumes of beer or cider per day, which means a weekly dose of more than 50 units [17]. The increased incidence of enamel erosion dependent on excess fluoride in a group of professional wine tasters is also reported [18, 19].

In summary the obtained results are difficult to interpret from the perspective of human health because of the differences in the fluoride level in these beverages depend on localization of manufacturing plants. Especially important is the content of fluoride in local water sources. But these data, as well as several reports by other authors, confirm the fact that alcoholic beverages need to be considered as a significant source of fluoride delivered into the body.
